# Infection dynamics of gastrointestinal helminths in sympatric non-human primates, livestock and wild ruminants in Kenya

**DOI:** 10.1371/journal.pone.0217929

**Published:** 2019-06-10

**Authors:** Vincent Obanda, Ndichu Maingi, Gerald Muchemi, Chege J. Ng’ang’a, Samer Angelone, Elizabeth A. Archie

**Affiliations:** 1 Department of Veterinary Services, Kenya Wildlife Service, Nairobi, Kenya; 2 Department of Pathology, Microbiology and Parasitology, Faculty of Veterinary Medicine, University of Nairobi, Nairobi, Kenya; 3 Estación Biológica de Doñana, Consejo Superior de Investigaciones Científicas (CSIC), Avda, Américo Vespucio s/n, Sevilla, Spain; 4 Institute of Evolutionary Biology and Environmental Studies (IEU), University of Zürich Winterthurerstrasse, Zürich, Switzerland; 5 Department of Biological Sciences, University of Notre Dame, South Bend, Indiana, United States of America; Universitat Autonoma de Barcelona, SPAIN

## Abstract

**Background:**

Gastrointestinal parasites are neglected infections, yet they cause significant burden to animal and human health globally. To date, most studies of gastrointestinal parasites focus on host-parasite systems that involve either a single parasite or a host species. However, when hosts share habitat and resources, they may also cross-transmit generalist gastrointestinal parasites. Here we explore multi-host-parasite interactions in a single ecosystem to understand the infection patterns, especially those linked to livestock-wildlife interfaces and zoonotic risk.

**Methods:**

We used both coprological methods (flotation and sedimentation; N = 1,138 fecal samples) and molecular identification techniques (rDNA and mtDNA; N = 18 larvae) to identify gastrointestinal parasites in nine sympatric host species (cattle, sheep, goats, wildebeest, Grant’s gazelles, Thomson’s gazelles, impala, vervet monkeys and baboons) in the Amboseli ecosystem, Kenya.

**Results:**

We found that the host community harbored a diverse community of gastrointestinal helminths, including 22 species and/or morphotypes that were heterogeneously distributed across the hosts. Six zoonotic gastrointestinal helminths were identified: *Trichuris* spp., *Trichostrongylus colubriformis*, *Enterobius* spp. *Oesophagostomum bifurcum*, *Strongyloides stercoralis* and *Strongyloides fuelleborni*. The dominant parasite was *Trichuris* spp, whose ova occurred in two morphological types. Baboons were co-infected with *Strongyloides fuelleborni* and *S*. *stercoralis*.

**Conclusions:**

We found that the interface zone shared by wild ungulates, livestock and non-human primates is rich in diversity of gastrointestinal helminths, of which some are extensively shared across the host species. Closely related host species were most likely to be infected by the same parasite species. Several parasites showed genetic sub-structuring according to either geography or host species. Of significance and contrary to expectation, we found that livestock had a higher parasite richness than wild bovids, which is a health risk for both conservation and livestock production. The zoonotic parasites are of public health risk, especially to pastoralist communities living in areas contiguous to wildlife areas. These results expand information on the epidemiology of these parasites and highlights potential zoonotic risk in East African savanna habitats.

## Background

Increased rates of incursion by people and livestock into wildlife areas, and the consequential interaction and exposure in the shared habitat, can have significant epidemiological consequences, especially for emerging infectious diseases, cross-transmission, and pathogen evolution [1–3]. Understanding parasite dynamics at such interfaces is therefore important to human and animal health, species conservation, and to designing effective prevention and control strategies against parasites [4–7]. In addition, human pastoralist communities share many environmental resources with domestic and wild animals, are at high risk of infection with zoonotic gastrointestinal parasites [8].

When diverse host species share resources in the same habitat, especially water and pasture, this sharing can influence contact rates and transmission of gastrointestinal parasites. This is because infective stages of these parasites are often fecally-dispersed on the environment and depend on both climatic and environmental conditions for propagation, persistence, and transmission (through ingestion) into hosts. However, the parasite communities that eventually get established in hosts are variable. A combination of host intrinsic and extrinsic traits, such as immune response, physiology, and behavior, influence the diversity of parasites that eventually establish in particular host species or populations [9, 10]. Thus, even when hosts live in the same habitat and are exposed to the same transmission opportunities, the potential community of parasites that are established among the hosts is likely variable, with some parasites predictably being dominant.

Several previous studies on parasite epidemiology focused on factors that are intrinsic to a single host or single host-parasite interactions, including habitat structure and ecology [11, 12], host behavior [13], host morphology [14] and host genetics [15–17]. Such studies have revealed a wealth of useful epidemiological information, but in nature, parasites typically occur as assemblages interwoven in a web of complex relationships with multiple hosts [18]. The effects of such intricate multi-host-parasite interactions are a subject of growing interest, especially the effects of co-infection on individual disease risk and health conditions [19–21].

The aim of this study is to explore parasitism in a naturally occurring multi-host species community, comprising livestock, wild ungulates and non-human primates using Amboseli ecosystem as a model. The Maasai pastoral community lives in this area, leading a transhumance way of life that exposes them to wildlife dominated habitats; exposure to both livestock and wildlife may present a public health risk, particularly by zoonotic gastrointestinal parasites. Unlike most previous studies on multi-host systems, which focus on closely related host species, we monitored a diverse and interactive animal community, including non-human primates (vervet monkeys and baboons), wild bovids (wildebeests, Grant’s gazelle, impala, and Thomson’s gazelle) and domestic ruminants (cattle, sheep, and goats). Given that non-human primates are phylogenetically closer to humans and both share many parasites, the parasite community of vervet monkeys and baboons in the present study could present public health risk to the pastoralist community in the study area.

## Methods

### Study area

The study was carried out in the Amboseli ecosystem, which includes Amboseli National Park (~392 km^2^) and the communally-owned Maasai group ranches (~5000 km^2^) that surround the National Park. The Amboseli ecosystem is a wildlife-livestock interaction zone; our study focused on livestock and wild mammals, including cattle (*Bos taurus*), sheep (*Ovis aries)*, goats (*Capra hircus*), impala *(Aepyceros melampus)*, Grant’s gazelle (*Nanger granti*), Thomson’s gazelle (*Eudorcas thomsonii*), wildebeest (*Connochaetes taurinus*), vervet monkeys (*Chlorocebus pygerythrus*), and baboons (*Papio cynocephalus*). Livestock are managed under a pastoralist system of husbandry. The ecosystem is characterized as a semi-arid savanna, with open grasslands mixed with patches of scrubs and *Acacia xanthophloea* woodlands. The Amboseli ecosystem receives highly variable annual rainfall that ranges between 141–757 mm, with an average annual rainfall of 340 mm (data available at http://amboselibaboons.nd.edu/). Natural springs and shallow wells dug by humans are the main sources of water for both livestock and wildlife.

### Coprological methods

#### Fecal sampling

Fecal samples for parasitological analyses were collected opportunistically from herds of wild and domestic ungulates and groups of non-human primates during both the wet season (January 2012) and the dry season (October 2012). Specifically, when we observed an individual animal defecating, we collected fresh dung from the ground that was not in direct contact with soil. In each season, within a 12-day period we collected 48 fecal samples each from cattle, goats, sheep, wildebeest, impala, Grant’s gazelles, Thomson’s gazelles, and vervet monkeys (N = 384 samples), while 185 samples were collected from baboons in each season (N = 185 baboon samples + 384 non-baboon samples = 569 samples per season across 2 seasons = 1,138 total samples). More samples were taken from baboons because this research was performed in collaboration with the Amboseli Baboon Research Project, which follows a population of wild, but habituated animals, making it very easy to collect many samples. Samples for coprological analysis were placed in labeled containers, pre-filled with buffered 10% formalin for preservation. In each season, we reserved a subset of 10 samples per host species to use in larval culture (N = 90 samples).

#### Fecal sedimentation and flotation

To search for parasites in fecal samples, fecal pellets were crushed and homogenized within the sampling container using a pestle. Sedimentation techniques was performed according to Vanderwaal et al 2014 [22]. Briefly, we weighed 3 grams of the fecal sample and added 45 mL of tap water to the sample in a 50 mL centrifuge tube. After stirring, the fecal slurry was sieved through a tea strainer and left to sediment for at least 10 minutes, after which the supernatant was gently decanted. This procedure was repeated until the suspension was clear. We pipetted 200μl of the sediment onto a glass slide and covered the sample with a cover slip (32 x 24 mm). We examined four slide preparations from each sediment sample under a light microscope (Leica DM500, Leica microsystems, UK) at 100X magnification.

To perform fecal flotation, we weighed 4 grams of homogenized fecal sample and mixed the sample with 12 mL of tap water. The fecal slurry was sieved through a tea strainer, and the filtrate was transferred to a 15 mL centrifuge tube. The sample was capped and centrifuged at 403 G for 10 minutes. The supernatant was decanted, and the sediment was re-suspended in Sheather’s flotation solution. The suspension was mixed and enough Sheather’s solution was added to form a slight bulging meniscus. We gently placed a cover slip on the meniscus and centrifuged the tubes for 10 minutes at 403 G. After centrifugation, we removed the cover slip, placed it on a glass slide and examined the slide at 100X magnification to identify and measure helminth eggs [23, 24]

### Molecular methods

#### Fecal culture and larval nematode isolation

To identify nematode parasites using molecular markers, we cultured larvae from a subset of fresh fecal samples following a modification of the Baermann technique [25]. Because of the labor and financial expense involved in genotyping from cultured larvae, only 10 samples per host in each season were subjected to larval culture. To perform fecal culture, samples from individuals of the same host species (within a season) were pooled into a single container. These pooled fecal samples were moistened with water and gently mixed. Approximately 10 g of the fecal mixture was placed into a plastic jar for culture at room temperature for 12 days. Fecal cultures were checked daily, moistened with water, and stirred to prevent fungal invasion.

On the 13^th^ day, we carried out larval extraction. The culture jars were filled with lukewarm water, stirred, and inverted on a petri dish. The exposed area of the petri dish around the inverted jar was filled with clean water and left to stand for 12 hours. Nematode larvae swam from the cultured fecal mixture into the clean water on the exposed part of the petri dish. The larval suspension (water containing the larvae) was examined under a stereo-microscope at 10X to 40X magnification. When a larva was located, it was collected and preserved in 95% ethanol. In total, we collected 977 individual larvae across all host species.

#### DNA extraction

To extract DNA, the ethanol was evaporated from each sample using a vacuum centrifuge. The dry larvae were lysed in 5 μL of PCR-quality water and 15μL of lysis buffer [2 μL 10X PCR Gold buffer (Fisher Scientific, New Jersey, USA; 0.8 μL Magnesium Chloride (Applied Biosystems, USA); 2 μL of 4.5% Nonidet P-40 (Amresco, Ohio, USA); 2 μL of 4.5% Tween 20 (Fisher Scientific, New Jersey, USA); 2 μL of a 2 mg/ml proteinase K (Applied Biosystems, USA) and 6.2 μL MilliQ water]. The sample was centrifuged briefly, and the larvae were freeze-fractured by placing them at -80 ^0^C for 20 minutes, followed by 100 minutes at 60 ^0^C and 20 minutes at 94 ^0^C. Because this method of DNA extraction can result in impure DNA containing PCR inhibitors, DNA extracts were diluted 3X in PCR-quality water and stored at -20 ^0^C.

#### Polymerase Chain Reaction (PCR)

Each DNA extract was amplified at two loci: (1) the region spanning ITS1, 5.8S and ITS2 of ribosomal DNA (rDNA), and (2) a portion of the mtDNA cytochrome oxidase 1 (CO1) gene of the mitochondrial DNA (mtDNA). The ITS locus is known to reliably differentiate closely related nematode species [26–32]. This marker is also popular due to its lower level of intra-species polymorphism compared to mtDNA [27, 33]. We further amplified CO1 because this locus undergoes rapid evolution and is good choice for differentiating cryptic parasite species [27] as well as phylogeographic groups within a single species [27, 33]. However, these genetic markers are rarely used together in a single study or for identification of a single nematode species yet when used in combination they enhance identification output and provide more genetic information [25, 26]. The ITS region was amplified using two sets of primers: (i) NC5 and NC2 and (ii) NC1and NC2 and amplified as previously published [26]. The CO1 gene was amplified using the primers LCO1490 and HCO2198 and protocol as previously published [34]. Gel electrophoresis (1.5% Agar, GP2 MIDSCI, USA) was used to detect positive amplifications.

#### Sequencing and sequence analysis

Positive PCR products were cleaned by ExoSAP and sent for Sanger sequencing at the Genomics unit of the University of Washington, Seattle, Washington, USA. The products were sequenced in both directions using Dye Terminator sequencing (Applied Biosystems). Sequence data were examined and cleaned using Sequencher software v. 4.10 (Gene codes corporation, Ann Arbor, Michigan USA). We searched these sequences for closest match to those in the GenBank using Basic Local Alignment Search Tool (BLAST) of the National Center for Biotechnology Information (NCBI). The sequences were deposited in the Genbank.

#### Phylogenetic analysis

We aligned sequences using ClustalX2 software [35] with the following alignment parameters: gap opening penalty at 15; gap extension at 6.66 and delay divergence set at 30%. We used MEGA 6.0 [36] to determine the best-fit nucleotide substitution model. Models with the lowest BIC scores (Bayesian Information Criterion) were considered to best describe the substitution patterns. All phylogenetic trees were derived by MEGA 6.0 based on the maximum likelihood method with 1000 bootstrap replicates.

#### Statistical analysis

A Student’s t-test was used to analyse the difference between parasite taxa in the wet and dry seasons.

#### Ethics statement

This research involved non-invasive coprological techniques and fecal samples used in this study did not necessitate the capture or disturbance to the study animals. Authorization and ethical clearance for this research was approved by the Research and Ethics Committee of the Kenya Wildlife Service (KWS/BRM/5001) and the University of Notre Dame (protocol number 16-10-3415).

## Results

**Fig 1:**
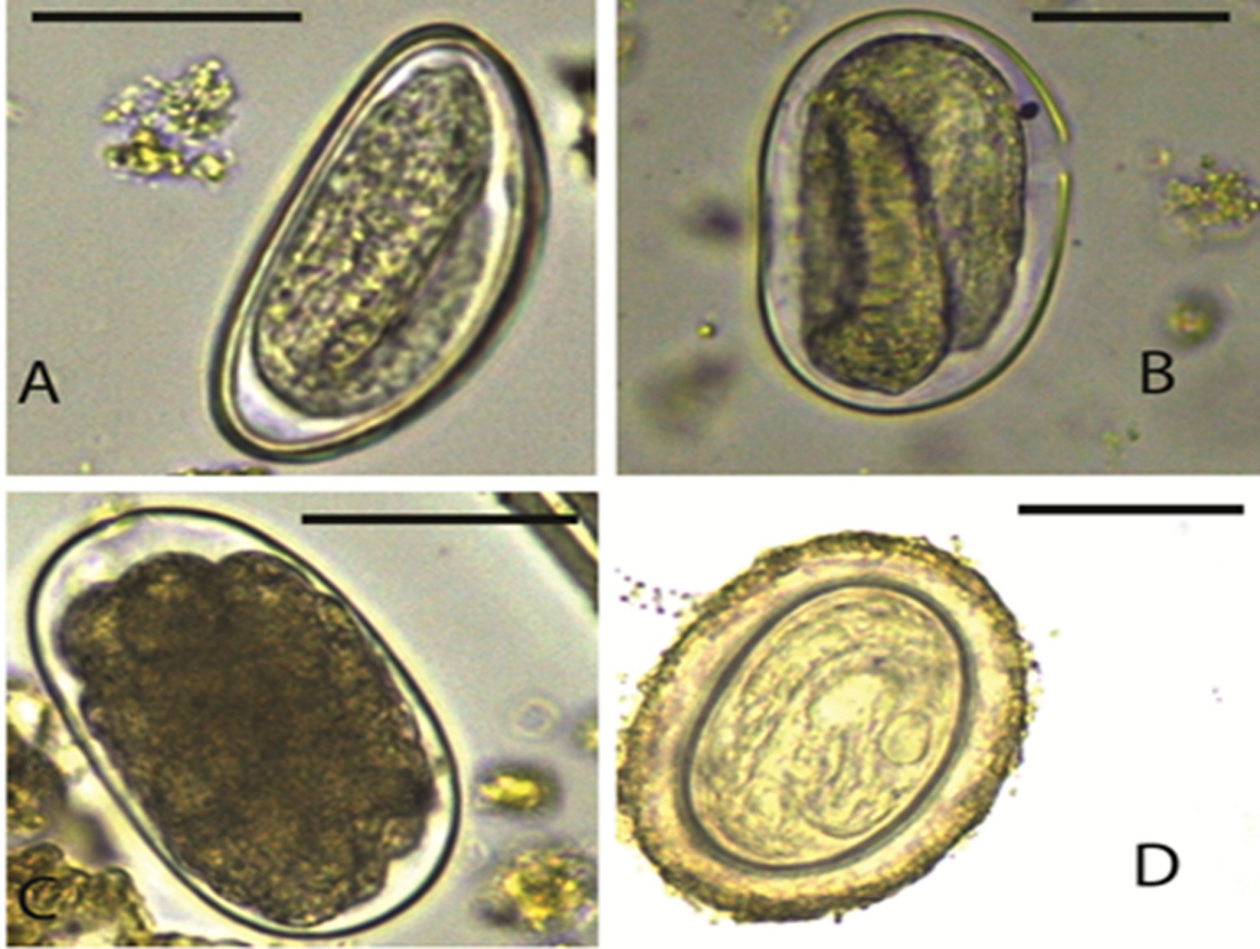
**Images of nematode eggs (A) *Enterobius* spp.; (B) *Strongyloides* spp.; (C) Strongylid type egg (D) *Primasubulura* spp.** Magnification x400, Scale 50 μm.

**Fig 2:**
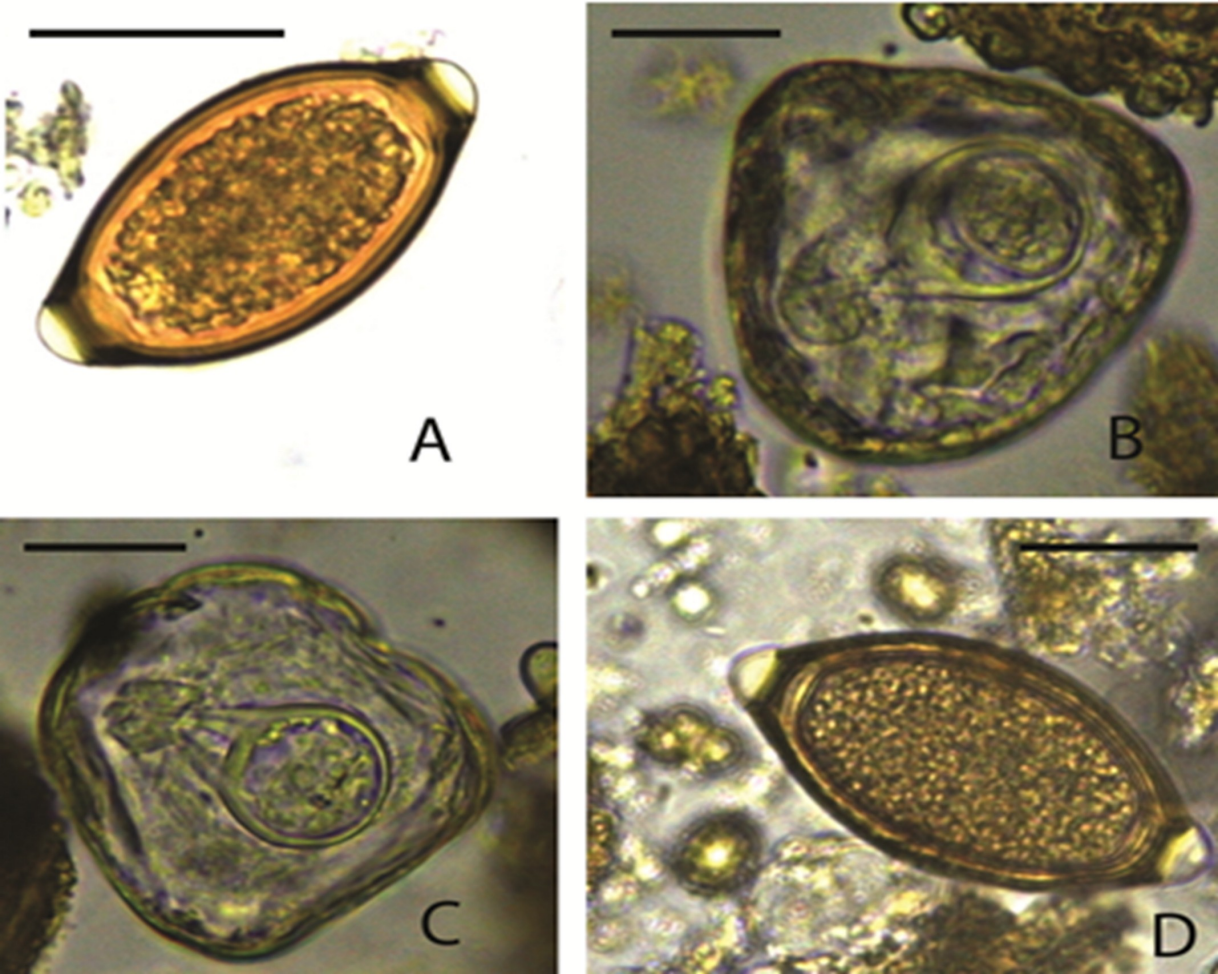
**Images of helminth eggs (A) *Trichuris* spp.*-OTU-d*; (B) *Moniezia expansa* (C) *Moniezia benedini* (D) *Trichuris* spp.*-OTU-c*.** Magnification x400, Scale 50 μm.

**Fig 3:**
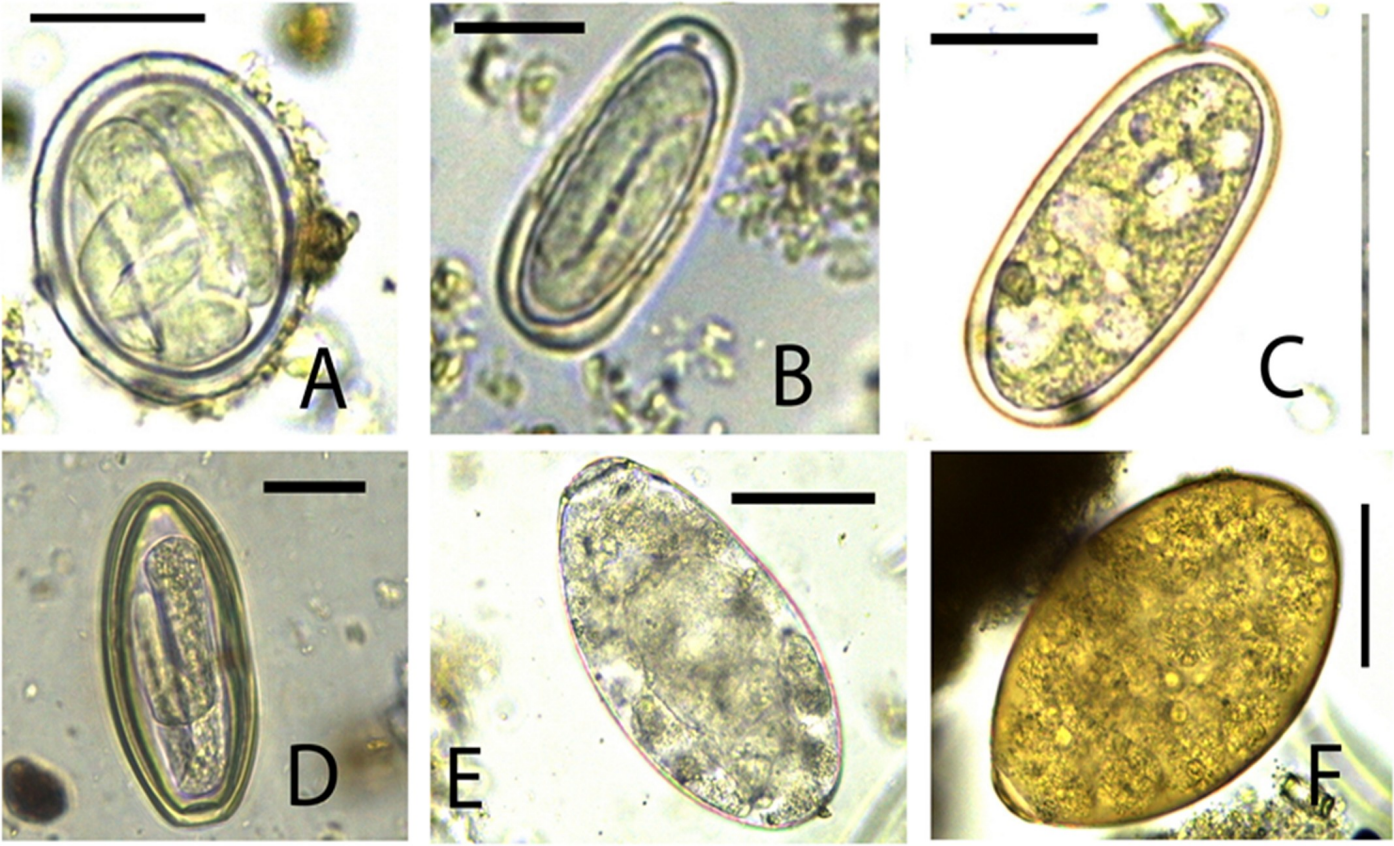
**Images of helminth eggs (A) *Spirurina* spp. (B) *Streptopharagus* spp. (C) Unidentified Spirurid*-*morphotype A. (D) Unidentified *Spirurid-*morphotype B (E) *Paramphistomum* spp. (F) *Fasciola hepatica*.** Magnification x400; Scale: 50 μm.

We identified a rich diversity of nematodes, trematodes and cestodes by egg morphology (Table 1) and molecular techniques. The total number of parasite taxa identified by both techniques was 22 (Table 2). Nematodes included *Enterobius* spp. (Fig 1A), *Strongyloides* spp. (Fig 1B), strongylid ova (Fig 1C), *Primasubulura* spp. (Fig 1D), *Trichuris* spp. (Figs 2A and 2D), *Spirurina* spp. (Fig 3A), *Streptopharagus* spp. (Fig 3B) and unidentified Spirurids (Fig 3C and 3D). The identified trematodes were *Paramphistomum* spp. (Fig 3E) and *Fasciola* spp. (Fig 3F). The cestodes were *Moniezia expansa* (Fig 2B) *and M*. *benedini* (Fig 2C). Within *Moniezia* spp. and *Trichuris* spp., we noted multiple egg morphotypes, which we categorized into operational taxonomic units (OTUs)—a proxy for grouping similar organisms, here specifically used to group nematode eggs with similar dimensions. These included *M*. *benedini* OTU A (92.2 by 79.2 μm) whose mean egg dimensions were larger compared to OTU B (61.6 by 58.4 μm), and *Trichuris* OTU C (73.5 by 36.1 μm) whose mean egg dimensions were larger than OTU D (60.1 by 29.9 μm). Based on morphological parasite identities, host species harbored significantly more helminth parasite taxa (t = 3.577, df = 8, p = 0.0072) in the wet season as compared to the dry season (Table 1). With the additional information provided by genetic markers, we found a total of 22 distinct parasite taxa (Table 2), with the highest parasite richness observed in non-human primates, followed by livestock and least in wild ungulates. Baboons exhibited the highest parasite richness; however, this difference was likely because of our greater sampling effort in baboons samples (we searched 185 baboon samples per season as compared to 48 samples for the other host species).

**Table 1:** Number of helminth taxa in the wet and dry seasons recovered by coprological methods in the animals in Amboseli ecosystem (Kenya).

Animal host	Number of helminth Taxa	
	Wet season	Dry season
**Cattle**	5	3
**Sheep**	3	2
**Goat**	5	2
**Wildebeest**	3	1
**Thomson’s gazelles**	4	2
**Grant’s gazelles**	2	2
**Impala**	1	1
**Vervet monkey**	5	5
**Baboon**	5	3
**Mean ± Std dev**	3.7 ± 1.5	2.3 ± 1.2

Note that we analyzed 48 samples from each host species in each season, with the exception of baboons, for which we analyzed 185 samples in each season.

**Table 2:** Parasite taxa identified from sympatric hosts using a combination of coprological and molecular methods.

Helminth diversity	Host species									Total
	Domestic ungulates			Wild ungulates				Non-human primates		
	Goat	Sheep	Cattle	Wildebeest	Thomson’s gazelle	Grant’s gazelle	Impala	Vervet monkey	Baboon	
*Oesophagostomum bifurcum*	x	x	x	x	x	x	x	x	√	1
*Trichostrongylus colubriformis*	x	x	x	x	x	x	x	x	√	1
*Cooperia oncophora*	x	x	√	x	x	x	x	x	x	1
*Haemonchus contortus*	√	x	x	x	x	x	x	x	x	1
*Teladosargia circumcincta*	x	x	x	x	x	√	x	x	x	1
*Moniezia benedini* (OTU-a)	x	x	√	√	x	x	x	x	x	2
*Moniezia benedini* (OTU-b)	√	√	x	x	x	x	x	x	x	2
*Moniezia expansa*	√	√	x	x	x	x	x	x	x	2
*Strongyloides* spp.	√	√	x	x	x	√	x	x	x	3
*Strongyloides stercoralis*	x	x	x	x	x	x	x	x	√	1
*Strongyloides fuelleborni*	x	x	x	x	x	x	x	x	√	1
Spirurid type i	x	x	x	x	x	x	x	x	√	1
Spirurid type ii	√	√	√	√	√	x	x	x	x	5
*Fasciola hepatica*	x	x	√	√	√	x	x	x	x	3
*Trichuris* spp. (OTU-c)	√	√	√	√	√	√	√	x	x	7
*Trichuris* spp. (OTU-d)	x	x	x	x	x	x	x	**√**	**√**	2
*Enterobius* spp.	x	x	x	x	x	x	x	**√**	**√**	2
*Spirurina* spp.	x	x	x	x	x	x	x	**√**	**√**	2
*Fasciola gigantica*	x	x	**√**	x	x	x	x	x	x	1
*Paramphistomum* spp.	x	x	**√**	x	x	x	x	x	x	1
*Primasubulura* spp.	x	x	x	x	x	x	x	√	√	2
*Streptopharagus* spp.	x	x	x	x	x	x	x	x	√	1
**Number of parasite taxa**	6	5	7	4	3	3	1	4	10	**43**
**Mean number of parasite taxa**	**6**			**2.75**				**7**		

Key: **√** denotes the presence of a helminth taxa; x denotes the absence of a helminth taxa.

Because of the challenges of genotyping cultured larvae (low DNA quantity and quality; see Methods), we only obtained positive PCRs from 285 reactions, and we were only able to obtain clean sequences, suitable for taxonomic identification, for 18 larvae. All of the identified larvae belonged to the families Trichostrongylidae (n = 12) and Strongyloididae (n = 6; Table 3).

**Table 3:** Nematode taxa isolated from wild and domestic host species in the Amboseli ecosystem (Kenya), which were identified using the mtDNA and ITS gene markers.

Identified helminths	Host species	Gene markers		
		cox1 gene	ITS gene	Total
*Strongyloides stercoralis*	Baboon	0	7	7
*Strongyloides fuelleborni*	Baboon	5	0	5
*Oesophagostomum bifurcum*	Baboon	0	1	1
*Trichostrongylus colubriformis*	Baboon	0	1	1
*Haemonchus contortus*	Goat	0	1	1
*Cooperia oncophora*	Cattle	0	2	2
*Teladosargia circumcincta*	Grant’s gazelle	0	1	1
Total		5	13	18

**Fig 4:**
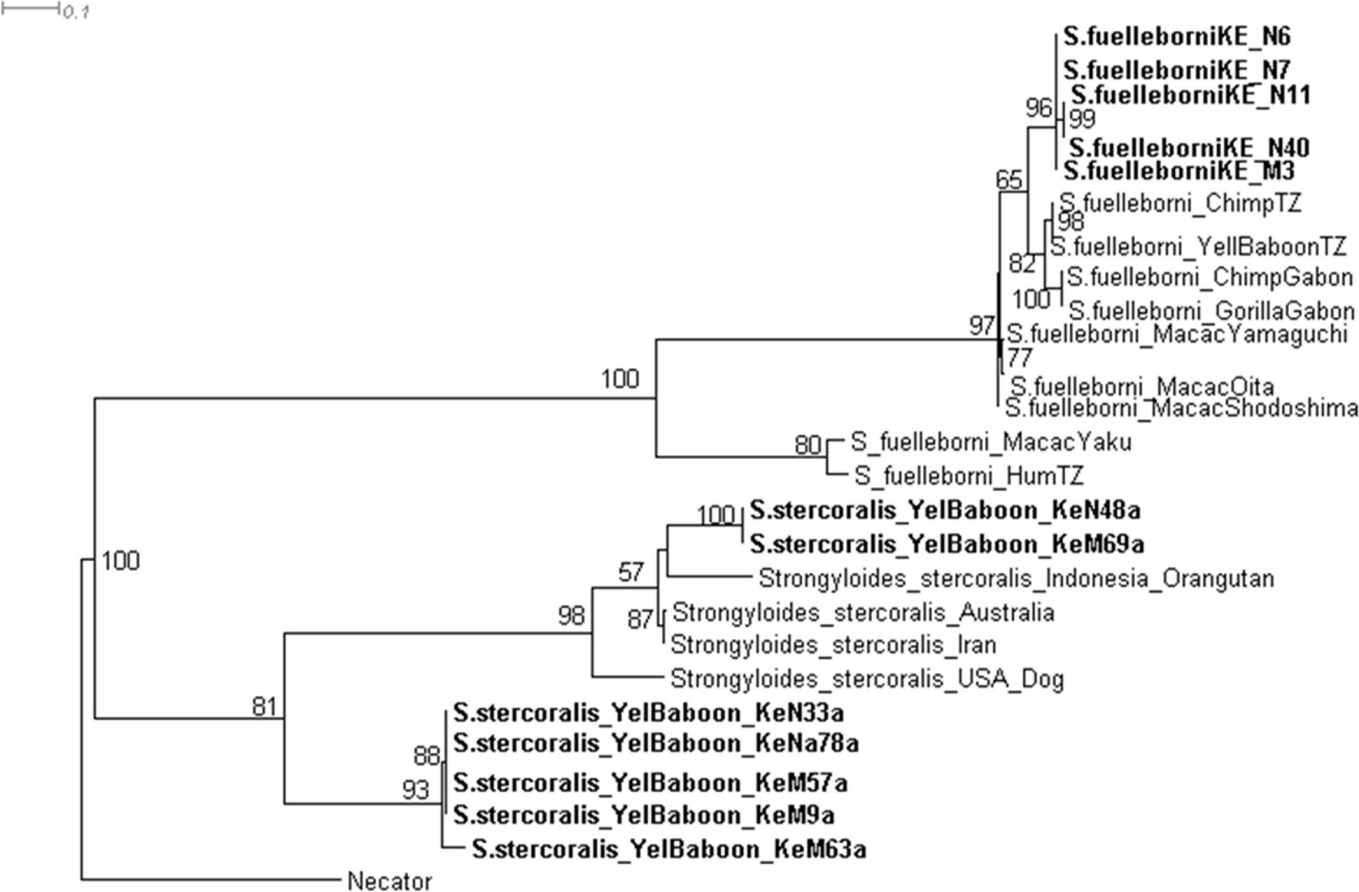
Evolutionary relationships of the isolates sequenced in this study (bold) with *Strongyloides* spp. sequences extracted from GenBank. The rooted maximum likelihood tree based on mtDNA was derived from 1000 bootstrap replicates using *Necator* spp. as outgroup. The numbers next to branches represent bootstrap values where values <50% were collapsed. Accession numbers for GenBank sequences included in the tree are in the S1 Table. Key: “Yelbaboon_Ke_” and “S.fuelleborniKE_” refers to the different isolates of *Strongyloides* spp. from yellow baboons in Kenya.

**Fig 5:**
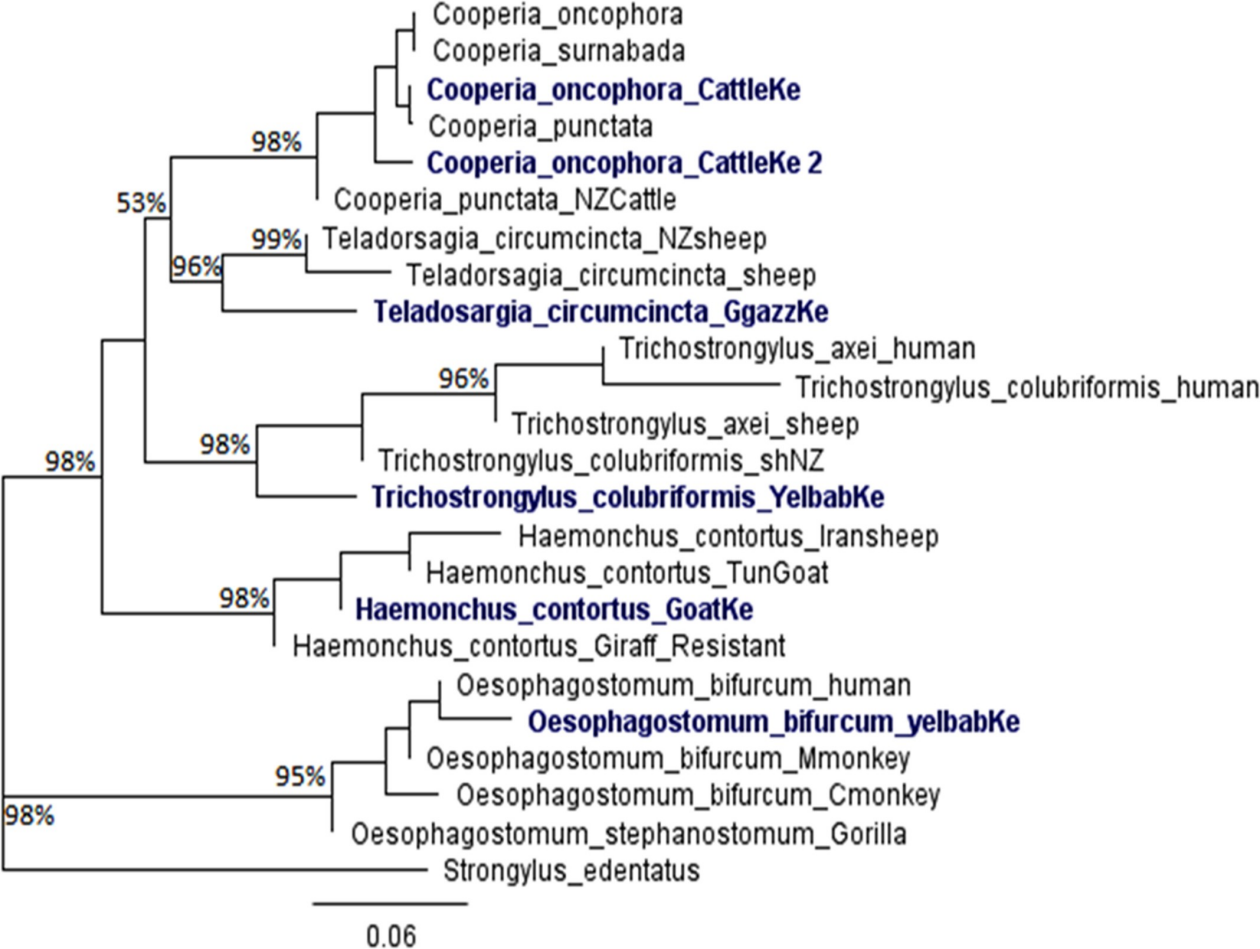
Evolutionary relationships of the isolates sequenced in this study (bold) with Trichostrongylid sequences selected from GenBank. The rooted maximum likelihood tree based on ITS of the rDNA was derived from 1000 bootstrap replicates using *Strongylus edentatus* as the outgroup. The numbers next to branches represent bootstrap values where values <50% were collapsed. Accession numbers for GenBank sequences included in the tree are in the S2 Table. Key: CattleKe- Cattle in Kenya; GgazzKe-Grant’s gazelle in Kenya; YelBabKe–Yellow baboon in Kenya; GoatKe–Goat in Kenya.

Molecular analysis revealed that baboons were infected with two species of *Strongyloides*: *S*. *fuelleborni* [GenBank: KT381715—KT381719] and *S*. *stercoralis* [GenBank: KT307984—KT307990] (Fig 4). Phylogenetic relationships among *S*. *fuelleborni* were structured according to the geographical location of their hosts rather than host taxonomic groups (Fig 4). All the sequences retrieved from the GenBank and used for the phylogenetic reconstruction of Fig 4 are in the S1 Table. We also identified several strongylid nematodes in hosts from Amboseli (Fig 5). Our single isolate of *Trichostrongylus colubrifomis* from Kenyan baboons [GenBank: KT215378] was distinct from *Trichostrongylus* sequences found on GenBank, which were sampled from humans and sheep (Fig 5). The *Oesophagostomum bifurcum* we identified from baboons [GenBank: KT215379] clustered with isolates on GenBank sampled from gorillas, humans, and other primates (Fig 5). Cattle and goats from Amboseli were infected by *Cooperia oncophora* [GenBank: KT215383] and *Haemonchus contortus* [GenBank: KT215381], respectively (Fig 5). We identified *Teladosargia circumcincta* [GenBank: KT215382] from Grant’s gazelle that clustered with GenBank isolates from sheep. Moreover, *Cooperia oncophora* clustered in a clade that included other isolates of *C*. *oncophora*, *C*. *surnabada*, and *C*. *punctata* (Fig 5). *Haemonchus contortus* had a closer relationship with other nematode species isolated elsewhere from goat sheep and giraffe. All the sequences retrieved from the GenBank and used for the phylogenetic reconstruction of Fig 5 are in S2 Table.

Combining information from our coprological and molecular methods, we identified distinct helminth taxa across the nine-host species (Table 2). All these sympatric host species were infected with at least one type of helminth, but helminth community composition varied across host species (Table 2). Molecular analysis revealed seven taxonomic groups of nematodes (Table 3) that could not have been differentiated by egg morphology. The single most dominant gastrointestinal parasite in the host community was *Trichuris* spp., occurring in all the nine-host species (Table 2). However, based on egg dimensions, morphotype *Trichuris* OTU C was only found in ungulates, while *Trichuris* OTU D was only found in baboons and vervet monkeys. Moreover, these two primate species shared four different species of helminths (*Primasubulura* spp., *Trichuris* spp., *Enterobius* spp and *Spirurina* spp).

## Discussion

This is the first study to assess parasite diversity in a sympatric host community that includes bovids (livestock and wild ungulates) and non-human primates. Our results reveal a dynamic parasite-host interaction characterized by parasite sharing among hosts, yet restricted by host evolutionary history. The combination of coprological and molecular tools has facilitated the detection of a rich diversity of parasites, including the differentiation of nematode species whose eggs are morphologically indistinguishable and often included in the generic category of ‘strongyle eggs’, which does not allow the exact parasite richness of the host to be known. The taxonomic diversity detected in the ungulates and primates from Amboseli includes species that are of veterinary and public health importance and whose epidemiology and phylogeography is less known in Kenya. In addition, we found that seasonality was linked to parasite richness such that hosts had higher diversity in the wet compared to dry season (Table 1). This pattern agrees with current scientific understanding of seasonal effects on helminth propagation, environmental persistence and transmission [37–39].

Further, in our study, genetic tools enabled us to differentiate closely related nematode species co-circulating within a single host population. The low number of genotyped larvae in this study was probably a consequence of multiple factors, such as loss of larvae during vacuum evaporation of ethanol, and poor DNA quality, which led to a high rate of sequencing errors. Therefore, in our opinion, the results of this study possibly do not represent the entire richness that is present in the nine host species studied in Amboseli.

The taxonomic richness of the parasites was highest in non-human primates, followed by livestock and least in wild ungulates (Table 2). Specifically, baboons harbored the highest number of distinct parasite species (Table 2), which is likely due to differences in parasite sampling intensity between hosts. We expected that wild bovids would harbor higher parasite richness compared to livestock because livestock in Amboseli are likely to be treated with anti-helmintics. However, contrary to this expectation, our results indicate that livestock in Amboseli are exposed to and infected by diverse helminth taxa that may be reciprocally transmitted between livestock and wild ruminants. It is generally expected that animals which occupy large home ranges or range widely should have higher prevalence and parasite species richness because they will likely encounter greater diversity of habitats and host taxa, which predispose them to higher risks of infection with more diverse parasites [40]. Although, the role of range size on parasite richness is not explicit and often inconclusive, we posit that the practice of transhumance exposes livestock to more diverse parasite infective stages leading to higher parasite diversity. In a previous study, cattle co-grazed with wildlife in a confined Conservancy in Kenya had lower parasite richness compared to the pastoralist cattle in the present study [22], which shows that range size used by the animal may influence parasite richness. Most of the wild bovids tend to have restricted range sizes, which could be a risk for higher worm intensity rather than diversity.

The presence of *Strongyloides* spp. in the host community is of public health interest because of the zoonotic nature of these nematodes. We found that the evolutionary relationships of *S*. *fuelleborni* were structured according to the geographical location of their hosts rather than host taxonomic groups (Fig 4). This observation agrees with Hasegawa *et al*. [41] indicates that *S*. *fuelleborni* populations in Kenya are genetically distinct from those in other countries, irrespective of whichever host they are identified from. However, evolutionary rates of *S*. *fuelleborni* found in different parts of Africa were apparently congruent (Fig 4), perhaps because these parasites are subject to similar selective forces. In contrast, rates of evolution of *S*. *fuelleborni* were dissimilar across locations in Japan (Fig 4). Specifically, *S*. *fuelleborni* isolates from populations of Macaque monkeys in Yaku Island were basal compared to those in Oita, Yamaguchi, and Shodoshima, a finding that may be caused by high levels of genetic drift in Japan (Fig 4).

We also observed that the baboon population in Amboseli was simultaneously infected by both *S*. *stercoralis* and *S*. *fuelleborni*. The phylogeny we reconstructed indicates that *S*. *stercoralis* is basal to *S*. *fuelleborni* (Fig 4). In addition, we observed a signal of *S*. *stercoralis* infection being structured by host species, which is comparable to *S*. *fuelleborni* (Fig 4). For instance, *S*. *stercoralis* in baboons was evolutionarily more like *S*. *stercoralis* from Orangutans, as compared to those from dogs and other host taxa (Fig 4). Information about *S*. *stercoralis* in Kenya is scarce because, to the best of our knowledge, only three surveys have been carried out since 1989 [42–44]; hence our results provide more insight to the epidemiology of this zoonotic parasite. Since *S*. *stercoralis* can infect humans by penetrating skin, and coupled with the fact that it can propagate inside humans (endogenous autoinfection), *S*. *stercoralis* is one of the most burdensome helminths causing long-term suffering [42, 45, 46]. In fact, over 100 million people worldwide suffer from *Strongyloides* spp.infections with *S*. *stercoralis* being the main cause [42, 47], though there are human cases of *S*. *fuelleborni* infections. People who frequently use or share habitats dominated by non-human primates typically acquire *S*. *fuelleborni* thought to be of a non-human primate origin [41], whereas *S*. *fuelleborni kellyi* is regarded as a human parasite restricted to Papua New Guinea [48]. In summary, the co-infections of *S*. *fuelleborni* and *S*. *stercoralis* in a baboon population show how closely related nematode species may vary in their genetic structuring and evolution, which implies that selection pressure is on the nematodes and not the host.

The presence of *Oesophagostomum bifurcum* in the Amboseli baboon population is also of concern from a public health perspective because of its zoonotic potential. The *O*. *bifurcum* we sampled from baboons was distinct from isolates found in other host species (Fig 5), which supports the theory of sub-structuring [49, 50]. Specifically, the sub-structuring theory posits that populations of *O*. *bifurcum* in baboons, Mona monkeys (*Cercopithecus mona*), Cynomolgus monkeys *(Macaca fascicularis)* and humans (Fig 5) differ in both morphology [51] and genetic sequences [49, 50]. The *O*. *bifurcum* we detected in baboons was much closer to isolates from humans (Fig 5), which may indicate greater potential for this parasite to infect both humans and baboons. Recent detection of multiple cryptic *Oesophagostomum* species that co-infect humans and other non-human primates [52] supports the existence of zoonotic *Oesophagostomum* spp. and therefore drives the need for more advanced genetic studies of this genus.

Baboons in the Amboseli ecosystem were previously found to harbor unidentified species of *Trichostrongylus* [53]; however, the genetic evidence in this study confirms that the baboons are infected with *T*. *colubriformis*. This nematode species that we identified in baboons was distinct from the rest of the *Trichostrongylus* spp. isolates on GenBank, including those identified from humans (Fig 5). Nematodes in the genus *Trichostrongylus* comprise many species of veterinary importance [54–56], and some species, such as *T*. *colubriformis*, are considered zoonotic. In areas where humans and animal hosts have overlapping habitats, *T*. *colubriformis* is a perennial public health burden [57–59]; so, our results suggest that this nematode also poses a potential risk to the local community in Amboseli. It is therefore important to determine actual human occurrence of *T*. *colubriformis* in the Amboseli community.

The genus *Haemonchus* comprises hematophagous trichostrongylid nematodes, of which *H*. *contortus* is the most widespread species, often associated with anaemia, livestock death, and huge economic and production losses in the livestock industry, especially in Africa [29, 60–62]. We identified *H*. *contortus* from goats only. A previous study suggests that *H*. *contortus* is highly prevalent among pastoralist goats in Kenya [63]. The *H*. *contortus* larvae found in goats clustered with other species identified elsewhere but was closer to those from goats rather than from sheep or giraffe, which may signify host clustering (Fig 5).

According to Sissay, Uggla [64], *T*. *circumcincta* is one of the most harmful strongylid nematodes in livestock, particularly sheep and goats in Africa. However, in the present study, *T*. *circumcincta* was only found in Grant’s gazelle. Presence of *T*. *circumcincta* in Grant’s gazelle is the first record in Kenya, but this species is not uncommon elsewhere in other gazelle species or livestock. The species of *T*. *circumcincta* from Grant’s gazelle were apparently distinct and more basal compared to species identified elsewhere from sheep (Fig 5). This may imply that the species identified in the gazelles have not undergone much genetic alterations as compared to those in sheep.

The genus *Cooperia* comprises Trichostrongyloid nematodes of veterinary importance as they contribute to mixed species helminthosis that leads to production losses in livestock worldwide [65, 66]. In the present study, *C*. *oncophora* was identified from the pastoralist cattle; the two isotypes identified indicates co-existence of within-population variation of *C*. *oncophora* of which one is distinct while the other is closer to *C*. *punctata*.

*Trichuris* spp. was the single most dominant parasite occurring in all the nine-host species. Based on egg size, there were two morphotypes of *Trichuris* structured according to host taxa. Specifically, there was the primate derived *Trichuris* spp. and the ungulate derived *Trichuris* spp. co-circulating in the host community (Table 2). Structural and dimensional variations in the morphology of nematode eggs are salient features used used as taxonomic proxy to group similar individuals for identification purpose [67]. Moreover, presence of variations in egg morphology (morphotypes) of a specific nematode may suggest deeper taxonomic cue [68] that may indicate cryptic species or genetic variations. For instance, recent genetic analysis of *Trichuris* spp. suggests the occurrence of multiple cryptic species that can infect both humans and non-human primates, while those infecting suids and other ungulates are distinct [55, 56, 69–71]. Hence, *T*. *trichiura* may not be the only cause of the half a billion people infected with Trichuriasis [72]: rather, this gastrointestinal disease likely includes multiple *Trichuris* species that cross-infect both humans and non-human primates. It is possible that the non-human primate derived *Trichuris* spp. in the Amboseli ecosystem is a public health risk to the pastoralist community who live in the area. So, there is need for an epidemiological study on the whipworm situation in the human-livestock-wildlife interface at the Amboseli ecosystem.

The intestinal cestode *Moniezia* spp. is of global occurrence, and its infection leads to economic losses in livestock, especially in calves and lambs. However, the taxonomy of *Moniezia* spp. is still unresolved, given that out of the seven putative species, genetic information is only available for three (*M*. *benedini*, *M*. *expansa* and *M*. *monardi*) [73, 74]. The adult worms and eggs of the two common *Moniezia* spp.: *M*. *benedini* and *M*. *expansa* are morphologically distinguishable, with *M*. *benedini* being a common infection in large domestic ruminants [75] as in the present study (cattle and wildebeest). In contrast, *M*. *expansa* are common in sheep and goats (Table 2). Our results, based on egg dimensions, indicate that *M*. *benedini* occurred in two morphotypes that were structured according to host species: we found one form of *M*. *benedini* (OTU A) in large ruminants, and another, (*M*. *benedini* OTU B) in small ruminants. This is the first report of occurrence of these morphotypes; existence of cryptic *Moniezia* species, especially within *M*. *benedini*, and is consistent with evidence provided by the multilocus enzyme electrophoresis [76].

Our results showed dichotomy in the infection pattern where by most of the parasites were shared among ruminants and *Trichuris* spp. was the only parasite shared between ruminants and non-human primates. Empirical and theoretical studies suggest that sharing of nematodes is more likely between closely related hosts e.g. bovids, [25, 77, 78] than hosts of distant ancestry. This is because closely related host species that share common ancestry are most likely to have similar ecology, physiology, behavioral traits that facilitate transmission, and immune repertoire against the parasite [16, 50]. For instance, strains of rabies virus are highly exchanged across closely related wild canine relatives [52], whereas the primate lentivirus is preferentially switched among closely related host species [53]. Among the non-human primates, our results showed that baboons and vervet monkeys shared four different species of helminths (*Primasubulura* spp., *Trichuris* spp., *Enterobius* spp., and *Spirurina* spp.), indicating that evolutionary similarity among hosts may predict similar parasite communities. The phylogenetic relatedness coupled by the geographic proximity between the non-human primates and people inhabiting the Amboseli ecosystem presents a risk for cross-transmission of the six zoonotic parasites and other pathogens. It is estimated that 45% of pathogens are shared by humans and non-human primates [79, 80]. Other non-human primate parasites; *Spirurina* spp. and *Primasubulura* spp. identified from the Amboseli population are not known to infect humans though they may still be of public health risk since many pathogens such as Simian Foamy Virus and Simian T-Lymphotropic Retroviruses that were restricted in non-human primates has of recent spilled-over into humans [81, 82]. It is argued that habitat sharing among multiple hosts is a major driver of emerging infectious diseases [83, 84] because pathogens spill-over and attain complete species-jumps into a new unusual host species, resulting in host shift, a process that involves adaptations and genetic changes [54, 55, 85–87].Host shift, which results into the expansion of host range for pathogens, is rarely reported in parasitic nematodes compared to those of viruses and bacteria since the latter have short generation turnover and rapid mutation rates [88, 89].

## Conclusion

It is clear that in a multi-species animal community like in the Amboseli ecosystem, parasite interaction with their hosts is so dynamic but also structured. For instance, some parasites infect multiple species across host taxa, while some tend to be restricted within hosts that are closely related. Clearly, bovids did not share gastrointestinal helminths with the non-human primates and even though *Trichuris* spp. was present in all the hosts, the morphotypes in bovids differed from those in non-human primates. In addition, we observed that each host had variable diversity of parasites, and unexpectedly, livestock had more diversity compared to wild ruminants. The bidirectional exchange of these parasites between wild ungulates and livestock is thus plausible and has potential implications on conservation and livestock productivity as well as anti-helminth control in this region. Most of the species found in non-human primates, *Trichuris* spp., *T*. *colubriformis*, *Enterobius*spp., *O*. *bifurcum*, *S*. *stercoralis* and *S*. *fuelleborni* are zoonotic and represent a potential risk to the pastoralist communities.

## Supporting information

S1 Table References for sequences of species of *Strongyloides* and *Necator* species selected from the Genebank and included in the phylogenetic tree. (DOCX)

S2 Table References for sequences of Trichostrongylids selected from Genebank and included in the phylogenetic tree. (DOCX)
