# Supplementary material for: Infection dynamics of gastrointestinal helminths in sympatric non-human primates, livestock and wild ruminants in Kenya
Source: PLoS One. 2019 Jun 10;14(6):e0217929. doi: 10.1371/journal.pone.0217929 (PMC6557494; doi:10.1371/journal.pone.0217929)
Supplement: S1 Table — (DOCX) [file pone.0217929.s001.docx]

**S1 Table**

| Nematode name | Host | Country | Gene | Accession number |
| --- | --- | --- | --- | --- |
| *Strongyloides stercoralis* | - | Australia | ITS | JX489154 |
| *Strongyloides stercoralis* | - | Iran | ITS | EF545004 |
| *Strongyloides stercoralis* | Orangutans | Indonesia | ITS | JF699149 |
| *Strongyloides stercoralis* | Dog | USA | ITS | U43962 |
| *Strongyloides fuelleborni* | Human | TZ/Japan | mtDNA | AB526282 |
| *Strongyloides fuelleborni* | Macaca | Japan | mtDNA | AB526291 |
| *Strongyloides fuelleborni* | Macaca | Japan | mtDNA | AB526293 |
| *Strongyloides fuelleborni* | Macaca | Japan | mtDNA | AB677957 |
| *Strongyloides fuelleborni* | Macaca | Japan | mtDNA | AB526290 |
| *Strongyloides fuelleborni* | Gorilla | Gabon | mtDNA | AB526289 |
| *Strongyloides fuelleborni* | Chimpanzee | Gabon | mtDNA | AB526288 |
| *Strongyloides fuelleborni* | Baboon | Tanzania | mtDNA | AB526285 |
| *Strongyloides fuelleborni* | Baboon | Tanzania | mtDNA | AB526306 |
| *Necator* spp. | Human | China | mtDNA | AJ417719 |
