# Supplementary material for: Infection dynamics of gastrointestinal helminths in sympatric non-human primates, livestock and wild ruminants in Kenya
Source: PLoS One. 2019 Jun 10;14(6):e0217929. doi: 10.1371/journal.pone.0217929 (PMC6557494; doi:10.1371/journal.pone.0217929)
Supplement: S2 Table — (DOCX) [file pone.0217929.s002.docx]

**S2 Table**

| Nematode name | Host | Country | Gene | Accession number |
| --- | --- | --- | --- | --- |
| *Oesophagostomum bifurcum* | Mona monkey | Australia | ITS | AF136575 |
| *Oesophagostomum bifurcum* | Human | - | ITS | Y11733 |
| *Oesophagostomum bifurcum* | *Macaca* | China | ITS | KF319024 |
| *Oesophagostomum stephanostomum* | Gorilla | Gabon | ITS | AB821022 |
| *Trichostrongylus colubriformis* | Sheep | New Zealand | ITS | KC998744 |
| *Trichostrongylus colubriformis* | Human | Thailand | ITS | KC337067 |
| *Trichostrongylus axei* | Sheep | Russia | ITS | EF427622 |
| *Teladorsagia circumcincta* | Sheep | New Zealand | ITS | KC998708 |
| *Teladorsagia circumcincta* | Sheep | Australia | ITS | X86026 |
| *Cooperia oncophora* | Sheep | Australia | ITS | X83561 |
| *Cooperia surnabada* | Sheep | Australia | ITS | AJ000032 |
| *Cooperia punctata* | Cattle | New Zealand | ITS | KC998744 |
| *Cooperia punctata* | Sheep | Australia | ITS | X83560 |
| *Haemonchus contortus* | Goat | Tunisia | ITS | JX901146 |
| *Haemonchus contortus* | Sheep | Iran | ITS | HQ389229 |
| *Haemonchus contortus* | Giraffe | USA | ITS | EU084689 |
| *Strongylus edentatus* | Equine | Australia | ITS | X77807 |
